# Affective temperaments show stronger association with infertility treatment success compared to somatic factors, highlighting the role of personality focused interventions

**DOI:** 10.1038/s41598-023-47969-x

**Published:** 2023-12-11

**Authors:** Georgina Szabo, Judit Szigeti F., Miklos Sipos, Szabolcs Varbiro, Xenia Gonda

**Affiliations:** 1 Doctoral School of Mental Health Sciences, Semmelweis University, Budapest, Hungary; 2 Institute of Behavioral Sciences, Semmelweis University, Budapest, Hungary; 3 Department of Obstetrics and Gynecology, Semmelweis University, Budapest, Hungary; 4 Department of Psychiatry and Psychotherapy, Semmelweis University, Budapest, Hungary; 5 NAP3.0-SE Neuropsychopharmacology Research Group, Hungarian Brain Research Program, Semmelweis University, Gyulai Pál Street 2, 1085 Budapest, Hungary

**Keywords:** Psychology, Outcomes research, Risk factors

## Abstract

Infertility has a multifactorial background, where, besides somatic factors, psychological contributors also play a role in development and outcome. While affective temperaments have been associated with development, course, and outcome as well as treatment success in various somatic conditions, their association with infertility and its treatment has not been investigated so far. The purpose of our retrospective cohort study was to evaluate the influence of affective temperaments on fertility treatment outcomes. Among 578 women who underwent infertility treatment in an Assisted Reproduction Centre in Budapest, Hungary, treatment success, detailed medical history, and demographic parameters were recorded, and the Temperament Evaluation of Memphis, Pisa, Paris, and San Diego Auto-questionnaire (TEMPS-A) was administered. Possible predictors of assisted reproduction outcome were analyzed using multivariate logistic regression models, followed by a receiver operating curve (ROC) analysis in order to define ideal affective temperament cut-off values for clinical applicability. Aside from age, BMI, and previous miscarriage, cyclothymic scores > 4 (OR = 0.51 CI 0.35–0.74, *p* < 0.001), depressive scores > 9 (OR = 0.59 CI 0.4–0.87, *p* = 0.009) and anxious scores > 9 (OR = 0.45 CI 0.31–0.66, *p* < 0.001) significantly decreased the odds of clinical pregnancy by 49%, 41% and 55%, respectively. Irritable and hyperthymic temperaments, as well as other somatic and socio-economic factors had no effect on infertility treatment outcomes. The results suggest that affective temperaments may be related to the outcome of infertility treatments. Thus, screening for affective temperaments may help identify high-risk patient groups and offer patient-tailored treatment, which may increase the chances of a successful pregnancy and live birth for women undergoing IVF treatment.

## Introduction

Affective temperaments (depressive, cyclothymic, hyperthymic, irritable, and anxious) represent the biological ‘cores’ of personality that manifest early and remain relatively stable throughout the lifespan^1^, and they define the individual's level of activity, basic mood, emotional reactivity, and related cognitions. In contrast to the majority of temperament models developed in psychology based on empirical research in infants focusing on healthy emotional reactivity, the affective temperament model was developed based on clinical and scientific observations of affective disorder patients and their unaffected first-degree relatives and has been validated in several clinical, theoretical, and molecular studies^2–6^. Although they have no pathological value in themselves, affective temperaments represent vulnerability factors for the development of various psychiatric^7,8^ and somatic conditions^9–20^, and potentially affect the long-term course and treatment outcome of these conditions^7,16,18,21^ either directly, via shared biological background factors, or indirectly, by influencing emotions, cognitions, and behaviors which may have an effect either on the development or the treatment of the illness^7,22^. In psychiatry affective temperaments especially when present in their marked or dominant form are also often considered high risk state for the development of affective disorders or even their subclinical or latent manifestations^23,24^, and beside robustly impacting risk of their development, are also useful for their characterisation, subtypisation, as well as in predicting long-term illness course^25^. Considering their strong etiological association with emotional reactivity and several behavioural and cognitive processes^5^, they are likely to be important determinants in case of somatic illnesses concerning risk of development, profile of symptomatic manifestation, longitudinal course, and they are also likely profoundly involved in various aspects of treatment from treatment seeking through adherence to outcome via divergent processes, as all of these components are underlied by emotional, cognitive, and behavioural processes^16,26,27^. There is recently increasing attention turning towards the involvement of psychological factors in the development and management of somatic illnesses in order to identify important targets or checkpoints for intervention, improving both outcomes and quality of life of patients, as well as potentially decreasing costs associated with missed prevention opportunities, late diagnosis, or high rates of unsuccessful treatment, residual symptoms, or side effects. Recently in a meta-analysis we demonstrated the strong involvement of affective temperaments in determining treatment adherence in both psychiatric and somatic patients^27^, however, the intricate and complex interrelationship between affective temperaments and course and outcome of somatic illnesses deserves further study to fully exploit its clinical implications. Understanding affective temperamental factors underlying divergent aspects of treatment and treatment outcomes may especially be important in case of interventions which are voluntary, involve a longer time course with strict adherence requirements potentially impacting daily life, show variable success rates, and are associated with a high emotional burden, and stress, such as infertility treatments^28^.

Infertility is a disease of the reproductive system, which, according to the World Health Organization, is defined as the failure to achieve pregnancy after 12 months or more of regular, unprotected sexual intercourse^29^. Infertility has been associated with psychological factors and has also been found to be underlied by various physiological conditions, somatic diseases^29^, and various lifestyle-related factors^30^, some of which may also be associated with affective temperaments^31–34^ which may thus directly or indirectly be involved in their development. On the other hand, via partially overlapping mechanisms, affective temperaments may also profoundly be involved in the success of infertility treatment by contributing to psychological status, compliance with infertility treatment and required lifestyle adjustments, and several other behaviors and cognitions that may influence treatment success in general and particularly in case of assisted reproduction. Therefore, affective temperaments may have a contributory role in the outcome of infertility and infertility treatments via both screening and predicting the likelihood of treatment success, as well as tailoring and personalizing treatment. Although affective temperaments, as biologically based personality traits that show remarkable stability throughout the lifespan may not be modifiable, they may define avenues for behavioral modification that may enhance treatment success. In spite of this, and the vast research on their role in the development and treatment of several somatic conditions, they have not been investigated in the context of infertility treatment outcome. The aim of the present study was to investigate the impact of affective temperaments on infertility treatment outcomes among women attempting assisted reproduction.

## Methods

### Study population

Hungarian women who requested their first appointment in the Assisted Reproduction Centre of the Department of Obstetrics and Gynecology, Semmelweis University, Budapest, Hungary, between November 2019 and November 2021 (n = 1773) were contacted retrospectively via email in November 2022. Out of them, n = 593 agreed to participate, n = 18 disagreed to participate, n = 1162 did not respond, the overall response rate being 34.5%. Inclusion criteria were as follows: women between 21 and 48 years (considering that assisted reproduction is supported in Hungary between the ages of 18 and 45), who have attempted fertility within the past 3 years, having good command of Hungarian language, willingness to participate in the study, providing informed consent, and completing the questionnaire.

Patients having fertility preservation due to cancer treatment or patients required assisted reproductive procedure solely due to known male but not female infertility, or patients visiting assisted reproduction centre with no intention of getting pregnant were excluded. Number of previous spontaneous or assisted pregnancies and number of children did not influence inclusion/exclusion in the study. Apart from this, all of the standard inclusion and exclusion criteria for participating in assisted reproduction procedures of course applied to our study sample. After removing duplicates and invalid records, a total of 578 participants were included in the study. Demographic, anthropometric, psychometric, and medical data of all patients were recorded by self-assessment questionnaires or were abstracted from the medical records. All patients agreed to data retrieval and analysis and provided written informed consent prior to filling the questionnaires. The study was approved by the Scientific and Research Ethics Committee of the Medical Research Council, the Hungarian Ministry of Health (IV/1568-1/2022/EKU) and was carried out in accordance with the tenets of the Declaration of Helsinki.

### Evaluation of affective temperaments

Affective temperaments were measured by the Temperament Evaluation of Memphis, Pisa, Paris and San Diego (TEMPS-A) auto-questionnaire. TEMPS-A is a 110-item self-report instrument, developed to assess affective temperaments in cyclothymic, depressive, anxious, irritable, and hyperthymic subscales, requiring “yes” (score 1) or “no” (score 0) answers^35,36^. The questionnaire showed good to excellent internal reliability of the scales in the Hungarian normative population in the validation study^36^: depressive (21 items, Cronbach’s alpha = 0.63); cyclothymic (21 items; Cronbach’s alpha = 0.81); irritable (seven items; Cronbach’s alpha = 0.79); anxious (26 items; Cronbach’s alpha = 0.84); and hyperthymic (21 items; Cronbach’s alpha = 0.78). In the current sample, very similar or better internal reliability of the scales was confirmed: depressive (21 items, Cronbach’s alpha = 0.66); cyclothymic (21 items; Cronbach’s alpha = 0.83); irritable (seven items; Cronbach’s alpha = 0.80); anxious (26 items; Cronbach’s alpha = 0.86); and hyperthymic (21 items; Cronbach’s alpha = 0.77).

### Assisted reproduction techniques

The techniques used were heterogeneous based on the patients' needs and associated medical conditions. In most cases, ovarian stimulation was applied according to one of the standard protocols^37^, followed by either traditional in vitro fertilization (IVF) or intracytoplasmic sperm injection (ICSI), supplemented as necessary with oral medication or other kind of treatment of the presumed problems behind the infertility. Due to the heterogeneity of the protocols and the fact that, according to a comprehensive meta-analysis, particular techniques have not been found to be predictors of treatment outcome^38^, in this study infertility treatment was considered a constant.

### Evaluation of treatment success

Infertility treatment success was defined as clinical pregnancy following infertility treatment, as reported by the patients.

### Evaluation of potential covariates

Age at the time of the first appointment was calculated from the difference between the date of the first appointment and the date of birth. BMI at the time of the first appointment was abstracted from the clinical records. Patients’ diagnosed somatic and psychiatric diseases were self-reported.

### Statistical analysis

Continuous variables are expressed as mean ± standard deviation (SD) and range, categorical variables are expressed as numbers and percentages. Between-group differences in descriptive characteristics and TEMPS-A scores were detected using the Wilcoxon rank sum test for continuous variables and Pearson's Chi-squared test or Fisher's exact test (when one or more cell counts were ≤ 5) for categorical values. TEMPS-A scores were compared to the normative population average using one-sample Wilcoxon signed rank test. To correct for multiple comparisons in each of the univariate analyses, Benjamini–Hochberg false discovery rate (FDR) adjusted p-values (q-values) were calculated. Calculating with a 5% false discovery rate, results with a q-value < 0.05 were considered significant.

Multivariate logistic regression analysis was used to determine the relationship between affective temperaments and infertility treatment success with all possible other predictors as covariates. The predictive power of affective temperaments was initially investigated as continuous variables. Since a high degree of intercorrelation is existent between affective temperaments, they were fit into the multiple regression analyses separately. Receiver operating curve (ROC) analysis was performed, and optimal cut-off values were defined based on the Youden index (sensitivity + specificity minus 1) for affective temperament scores in each of the five subscales. The results are presented as odds ratios (OR) and 95% confidence intervals (CI). The nominal significance threshold was p < 0.05 in all analyses. All calculations were performed using R Statistical Software (Vienna, Austria version 4.2.2).

## Results

In total, our retrospective cohort study included 578 women (22–46 years of age) who underwent infertility treatment in the Assisted Reproduction Centre of the Department of Obstetrics and Gynecology of Semmelweis University in Budapest, Hungary, between November 2019 and November 2021. 366 (63%) of the participants were primary infertile, 84 (15%) already had at least one child from a previous pregnancy, while 128 (22%) did not have children yet but already achieved to get pregnant which ended in miscarriage(s). The mean age of our cohort was 35.78 ± 4.74 years, and the mean BMI 24.24 ± 4.90 kg/m^2^. In terms of affective temperaments, the current infertile population differed from the average obtained in the otherwise smaller normative sample (n = 438), as the cyclothymic, anxious and irritable average scores were significantly lower than the Hungarian female population average examined by Rozsa et al. (4.43 ± 3.94 vs 7.98 (W = 21,618, q = 0.005), 7.21 ± 5.22 vs 8.06 (W = 60,484, q = 0.005), and 4.02 ± 3.52 vs 5.88 (W = 38,927, q = 0.005), respectively)^36^. Depressive and hyperthymic average scores were not significantly different compared to the population mean (7.3 ± 3.03 vs 7.35 (W = 79,754, q = 0.486), and 10.15 ± 4.04 vs 10.29 (W = 81,423, q = 0.700), respectively).

The probable causes of infertility were highly variable among the patients, the most typical of which included various problems of carbohydrate metabolism (42%), thyroid function problems (36%), endometriosis (10%), or a combination of these, while in case of 51% of the patients there was no identifiable disease behind infertility. The applied assisted reproduction treatments were also diverse. In most cases ovarian stimulation was applied according to one of the standard protocols^37^ followed by either traditional in vitro fertilization (IVF) or intracytoplasmic sperm injection (ICSI), supplemented as necessary with medications or other kinds of treatment for the presumed problems behind the infertility. The applied infertility treatment resulted in clinical pregnancy in 225 out of 578 cases (39%).

The mean age of patients who successfully became pregnant after the treatment was approximately 2 years less, as compared to those who have failed to conceive after treatment (34.43 ± 4.47 vs 36.65 ± 4.72 years (W = 50,414, *q* = 0.005)). No statistically significant difference could be observed regarding BMI, nor socio-economic parameters as education or residence, nor regarding clinical history parameters as previous pregnancy, miscarriage, live birth, known psychiatric or somatic illnesses, or the time since being diagnosed with infertility.

Regarding affective temperaments, the mean score of cyclothymic and anxious temperaments proved to be significantly lower in the pregnant cohort compared to the non-pregnant one (3.80 ± 3.64 vs 4.83 ± 4.08 points (W = 45,689, *q* = 0.008) and 6.45 ± 4.81 vs 7.69 ± 5.42 points (W = 44,772, *q* = 0.035), respectively). Demographic parameters, clinical data, and scores in the different scales of affective temperaments are summarized in Table 1.

**Table 1:** Demographic parameters, infertility risk factors and TEMPS-A scores in patients with successful and unsuccessful infertility treatment.

Characteristics	Total	Treatment success (clinical pregnancy)		Test statistic	p-value	q-value
		(−)	(+)			
Number	578	353 (61%)	225 (39%)	–	–	–
Age (years)	35.78 (4.74) [22.00, 46.00]	**36.65 (4.72)** **[22.00, 45.00]**	**34.43 (4.47)** **[24.00, 46.00]**	W = 50,414	< 0.001	**0.005**
BMI (kg/m^2^)	24.24 (4.90) [16.50, 55.60]	24.61 (5.24) [16.50, 55.60]	23.66 (4.27) [17.00, 42.40]	W = 43,276	0.069	0.149
Affective temperaments						
Cyclothymic	4.43 (3.94) [0.00, 19.00]	**4.83 (4.08)** **[0.00, 18.00]**	**3.80 (3.64)** **[0.00, 19.00]**	W = 45,689	0.002	**0.008**
Depressive	7.30 (3.03) [0.00, 19.00]	7.56 (3.12) [0.00, 19.00]	6.89 (2.83) [0.00, 18.00]	W = 44,120	0.024	0.075
Anxious	7.21 (5.22) [0.00, 23.00]	**7.69 (5.42)** **[0.00, 23.00]**	**6.45 (4.81)** **[0.00, 22.00]**	W = 44,772	0.01	**0.035**
Irritable	4.02 (3.52) [0.00, 16.00]	4.03 (3.47) [0.00, 16.00]	4.00 (3.61) [0.00, 16.00]	W = 40,524	0.7	0.754
Hyperthymic	10.15 (4.04) [0.00, 21.00]	9.89 (4.05) [0.00, 21.00]	10.55 (3.99) [0.00, 20.00]	W = 35,964	0.055	0.128
Clinical history						
Infertile for (years)				χ^2^ = 3.2	0.075	0.150
< 2	302 (52%)	174 (49%)	128 (57%)			
> 2	276 (48%)	179 (51%)	97 (43%)			
Previous pregnancy	212 (37%)	127 (36%)	85 (38%)	χ^2^ = 0.19	0.7	0.754
Previous miscarriage	197 (34%)	114 (32%)	83 (37%)	χ^2^ = 1.3	0.3	0.467
Previous live birth	84 (15%)	52 (15%)	32 (14%)	χ^2^ = 0.03	0.9	0.900
Psychiatric illness	221 (38%)	142 (40%)	79 (35%)	χ^2^ = 1.5	0.2	0.350
Chronic disease (any kind)	285 (49%)	170 (48%)	115 (51%)	χ^2^ = 0.48	0.5	0.667
Metabolic disorder	243 (42%)	149 (42%)	94 (42%)	χ^2^ = 0.01	> 0.9	0.900
Thyroid disorder	206 (36%)	123 (35%)	83 (37%)	χ^2^ = 0.25	0.6	0.700
Endometriosis	59 (10%)	33 (9.3%)	26 (12%)	χ^2^ = 0.73	0.4	0.560
Socio-economic factors						
Education				Fisher’s ET	0.2	0.350
Primary	5 (0.9%)	4 (1.1%)	1 (0.4%)			
Secondary	437 (76%)	258 (73%)	179 (80%)			
Tertiary	136 (24%)	91 (26%)	45 (20%)			
Residence				χ^2^ = 2.2	0.3	0.467
Capital	273 (47%)	163 (46%)	110 (49%)			
City	72 (12%)	40 (11%)	32 (14%)			
Town, village	233 (40%)	150 (42%)	83 (37%)			

Continuous variables are expressed as mean ± standard deviation (SD) and range, categorical variables are expressed as numbers and percentages. The p-values are calculated by Wilcoxon rank sum test, Pearson's Chi-squared test or Fisher's exact test. The q values represent false discovery rate correction (adjusted p value) for multiple testing. The bold values in the table represent significant findings. BMI: body mass index; Fisher’s ET: Fisher's exact test.

Logistic regression showed that cyclothymic, depressive, and anxious temperaments were associated with infertility treatment success in the regression models corrected for age, BMI, socio-economic and clinical history parameters. Cyclothymic (odds ratio (OR) = 0.91 CI 0.86–0.96, *p* = 0.001), depressive (OR = 0.91 CI 0.86–0.97, *p* = 0.006) and anxious (OR = 0.94 CI 0.9–0.97, *p* = 0.001) affective temperaments significantly decreased the odds of clinical pregnancy. Apart from affective temperaments, only age, BMI and previous miscarriage were shown to be predictors of infertility treatment success. Table 2 presents the results of the logistic regression analyses.

**Table 2:** Results of logistic regression analyses of possible predictors of assisted reproduction treatment success (clinical pregnancy).

Predictors	Model 1			Model 2			Model 3			Model 4			Model 5		
	OR	95% CI	p	OR	95% CI	p	OR	95% CI	p	OR	95% CI	p	OR	95% CI	p
Age	**0.87**	**0.83–0.91**	** < 0.001**	**0.87**	**0.84–0.91**	** < 0.001**	**0.87**	**0.83–0.91**	** < 0.001**	**0.88**	**0.84–0.91**	** < 0.001**	**0.88**	**0.84–0.92**	** < 0.001**
BMI	0.96	0.92–1	0.059	**0.95**	**0.91–0.99**	**0.019**	**0.96**	**0.92–1**	**0.032**	**0.96**	**0.92–0.99**	**0.030**	**0.95**	**0.91–0.99**	**0.019**
Cyclothymic	**0.91**	**0.86–0.96**	**0.001**												
Depressive				**0.91**	**0.86–0.97**	**0.006**									
Anxious							**0.94**	**0.9–0.97**	**0.001**						
Irritable										0.99	0.94–1.05	0.825			
Hyperthymic													1.03	0.99–1.08	0.157
Infertile for (> 2 years)	0.96	0.66–1.4	0.843	0.97	0.67–1.41	0.888	0.96	0.66–1.39	0.822	0.92	0.64–1.34	0.675	0.93	0.64–1.34	0.699
Previous pregnancy	0.89	0.47–1.67	0.709	0.90	0.48–1.68	0.733	0.91	0.48–1.72	0.779	0.97	0.52–1.81	0.927	0.97	0.52–1.81	0.924
Previous miscarriage	**2.17**	**1.2–3.96**	**0.011**	**1.97**	**1.1–3.54**	**0.023**	**2.06**	**1.15–3.74**	**0.016**	**1.89**	**1.06–3.37**	**0.031**	**1.84**	**1.03–3.28**	**0.038**
Previous live birth	1.25	0.67–2.3	0.484	1.21	0.66–2.24	0.535	1.21	0.65–2.23	0.540	1.16	0.63–2.13	0.620	1.17	0.64–2.14	0.608
Psychiatric illness	0.89	0.6–1.32	0.558	0.85	0.58–1.26	0.421	0.87	0.59–1.29	0.499	0.74	0.5–1.09	0.131	0.77	0.52–1.12	0.168
Chronic disease	1.32	0.89–1.96	0.176	1.30	0.87–1.93	0.197	1.34	0.9–2.01	0.148	1.23	0.83–1.82	0.304	1.25	0.84–1.85	0.272
Metabolic disorder	0.99	0.67–1.45	0.942	1.00	0.68–1.46	0.984	0.99	0.67–1.46	0.968	0.96	0.65–1.4	0.821	0.98	0.66–1.43	0.901
Thyroid disorder	1.09	0.74–1.62	0.656	1.09	0.74–1.61	0.656	1.08	0.73–1.6	0.692	1.10	0.75–1.62	0.627	1.10	0.75–1.63	0.620
Endometriosis	1.08	0.58–1.99	0.802	1.04	0.57–1.91	0.892	1.05	0.57–1.93	0.863	1.01	0.55–1.85	0.966	1.01	0.55–1.84	0.973
Education (primary)	–	–	–	–	–	–	–	–	–	–	–	–	–	–	–
Education (secondary)	4.55	0.52–99.8	0.216	3.55	0.42–78.74	0.300	4.86	0.57–109.4	0.200	4.21	0.51–91.42	0.234	4.07	0.51–87.04	0.240
Education (tertiary)	5.96	0.7–130.4	0.143	4.87	0.58–107.9	0.195	6.81	0.81–153.2	0.119	5.98	0.74–129.3	0.138	5.71	0.72–121.64	0.144
Residence (capital)	–	–	–	–	–	–	–	–	–	–	–	–	–	–	–
Residence (city)	1.30	0.74–2.3	0.360	1.25	0.71–2.21	0.432	1.27	0.72–2.22	0.411	1.20	0.68–2.1	0.526	1.19	0.68–2.08	0.544
Residence (town, village)	0.85	0.57–1.26	0.414	0.81	0.54–1.19	0.284	0.84	0.57–1.25	0.395	0.79	0.54–1.17	0.245	0.81	0.55–1.21	0.305

The table shows the results of multivariate logistic regression analyses using age, BMI, change in TEMPS-A scores, socio-economic and clinical history parameters as predictor and clinical pregnancy as outcome variables. Affective temperaments were fit into the multiple regression analyses separately. OR odds ratio, CI confidence interval, BMI body mass index. The bold values in the table represent significant findings.

Based on multiple ROC analyses, cyclothymic scores > 4 [sensitivity: 55.8%; specificity: 60.8%; AUC (Area Under the Curve): 0.575], depressive scores > 9 (sensitivity: 33.4%; specificity: 74.2%; AUC: 0.556), anxious scores > 9 (sensitivity: 40.2%; specificity: 73.3%; AUC: 0.564), irritable scores > 3 (sensitivity: 60.1%; specificity: 44.9.2%; AUC: 0.510), and hyperthymic scores > 8 (sensitivity: 78.7%; specificity: 30.0%; AUC: 0.547) yielded the highest Youden index and were thus defined as optimal cut-off values. The distribution of TEMPS-A categories defined by these optimal cut-off values in the overall patient population, as well as among patients with successful and unsuccessful infertility treatment are described in Table 3.

**Table 3:** Distribution of TEMPS-A categories in the overall patient population, and among patients with successful and unsuccessful infertility treatment.

Characteristics	Total	Treatment success (clinical pregnancy)		Test statistic	p-value	q-value
		(−)	(+)			
Number	578	353 (61%)	225 (39%)	–	–	–
Affective temperaments						
Cyclothymic > 4	240 (42%)	**166 (47%)**	**74 (33%)**	χ^2^ = 11	** < 0.001**	**0.005**
Depressive > 9	176 (30%)	118 (33%)	58 (26%)	χ^2^ = 3.8	0.051	0.128
Anxious > 9	202 (35%)	**142 (40%)**	**60 (27%)**	χ^2^ = 11	** < 0.001**	**0.005**
Irritable > 3	263 (46%)	164 (46%)	99 (44%)	χ^2^ = 0.34	0.6	0.700
Hyperthymic > 8	374 (65%)	217 (61%)	157 (70%)	χ^2^ = 4.1	0.042	0.118

Categorical variables are expressed as numbers and percentages. The p-values are calculated by Pearson's Chi-squared test. The q values represent false discovery rate correction (adjusted p value) for multiple testing. The bold values in the table represent significant findings.

**Figure 1:**
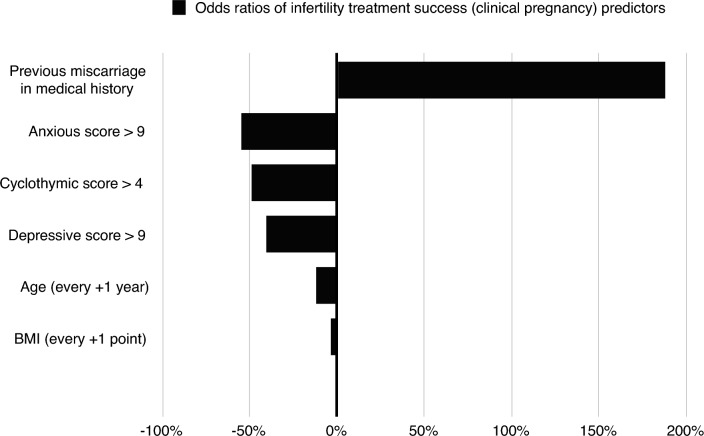
Statistically significant predictors of infertility treatment success.

According to further logistic regression analyses, cyclothymic scores > 4 (OR = 0.51 CI 0.35–0.74, *p* < 0.001), depressive scores > 9 (OR = 0.59 CI 0.4–0.87, *p* = 0.009) and anxious scores > 9 (OR = 0.45 CI 0.31–0.66, *p* < 0.001) independently predict infertility treatment success (clinical pregnancy), along with age, BMI, and previous miscarriage. More specifically, a cyclothymic score > 4, depressive score > 9 and anxious score > 9 decreases the odds of clinical pregnancy after infertility treatment by 49%, 41% and 55% respectively (Fig. 1). Table 4 summarizes the results of the final logistic regression analyses.

**Table 4:** Results of logistic regression analyses applying optimal cut-off values for affective temperaments.

Predictors	Model 1			Model 2		Model 3			Model 4			Model 5			
	OR	95% CI	p	OR	95% CI	p	OR	95% CI	p	OR	95% CI	p	OR	95% CI	p
Age	**0.88**	**0.84–0.92**	** < 0.001**	**0.88**	**0.84–0.91**	** < 0.001**	**0.87**	**0.84–0.91**	** < 0.001**	**0.88**	**0.85–0.92**	** < 0.001**	**0.89**	**0.85–0.92**	** < 0.001**
BMI	**0.96**	**0.92–1**	**0.036**	**0.95**	**0.92–0.99**	**0.012**	**0.95**	**0.92–0.99**	**0.013**	**0.95**	**0.92–0.99**	**0.012**	**0.95**	**0.91–0.98**	**0.007**
Previous miscarriage	**1.88**	**1.27–2.79**	**0.002**	**1.77**	**1.2–2.61**	**0.004**	**1.87**	**1.26–2.77**	**0.002**	**1.77**	**1.2–2.61**	**0.004**	**1.70**	**1.16–2.51**	**0.007**
Cyclothymic	**0.51**	**0.35–0.74**	** < 0.001**												
Depressive				**0.59**	**0.4–0.87**	**0.009**									
Anxious							**0.45**	**0.31–0.66**	** < 0.001**						
Irritable										0.84	0.59–1.2	0.336			
Hyperthymic													1.44	1–2.1	0.054

The table shows the results of multivariate logistic regression analyses using age, BMI, previous miscarriage, and TEMPS-A scores with optimal cut-off values applied as predictor and clinical pregnancy as outcome variables. Affective temperaments were fit into the multiple regression analyses separately. OR odds ratio, CI confidence interval, BMI body mass index. The bold values in the table represent significant findings.

## Discussion

Our study revealed that, aside from traditional risk factors, cyclothymic, depressive, and anxious affective temperaments are independent predictors of infertility treatment success. Higher cyclothymic, depressive, and anxious temperament scores decreased the odds of clinical pregnancy following infertility treatment in our cohort of 578 infertile patients. Irritable and hyperthymic temperaments had no effect on treatment success. We also found that, besides known predictors of infertility treatment success, such as age, BMI, or previous miscarriage^39–41^, other somatic and socio-economic factors have no or minimal effect on infertility treatment outcomes, their effect, if present, being much smaller compared to that of affective temperaments.

We are increasingly aware of how personality and psychological factors have a marked effect on several somatic diseases, not only on their development, but also their long-term course and the success of their treatment. Affective temperaments may directly and indirectly contribute to these via several potential mechanisms^16,18,21,31,32,42–46^.

Regarding the relationship between psychology and infertility, different models have evolved since the 1940s^47^, starting with the since outdated psychogenic model, which considered psychopathology and unconscious psychological processes as the sole causes of biologically unexplained infertility^48^, followed by various psychosomatic models, which likewise focus on the psychological contributors of the development of infertility^47^, the most intensively researched of which is the stress hypothesis, which examines the association between neuroendocrine sequelae of chronic stress and fertility problems^49^. Stress was found to be associated with fluctuations in blood sugar levels, insulin resistance, obesity, hypothyroidism, or hyperprolactinemia, all of which may complicate achieving pregnancy^50,51^, and some of which somatic conditions have already been associated with affective temperaments^20,31,32,42,52,53^. More recent results also suggest that the effects of stress may not be mediated by the above-mentioned hormonal-biological factors, but instead operate through deterioration of health behaviors, some of which are also associated with affective temperaments^54,55^, such as smoking, alcohol intake, drug use and lack of physical activity^30^. State-of-the-art approaches to the psychological background of infertility also consider cognitions or behaviors reducing the chance of conception, for example, by limiting sexual intercourse to infertile days, improper nutrition, smoking, drug consumption, competitive sports or extreme work stress, or premature stopping of fertility treatments^56,57^. Some of these behaviours, such as adherence to doctor’s recommendations^27^ or smoking^54^ have already been reported to be influenced by affective temperaments.

Another group of psychological models of infertility considers psychological distress as a consequence rather than a cause of infertility^47^, and they examine the role of psychological factors in the treatment instead of the development of infertility. These models draw attention to depression and anxiety caused by infertility due to constant stress, disappointment, and accumulated loss^58^, which, in turn, further worsen the chances of pregnancy. Also, they focus on psychological screening prior to infertility treatment to identify patients at risk of emotional problems during treatment^59,60^, study the means and effectiveness of different types of psychological assistance supporting treatment^61,62^, and, most recently, psychological factors for non-adherence to treatment protocol and early drop-out^63,64^. Moreover, very importantly, studies also examined the effect of couple-related psychological factors on the success of the assisted reproduction procedure, including couple relationship, type of romantic attachment, and fertility-related quality of life^65^.

Regarding affective temperaments, they have not only been directly associated with depression and anxiety, but also with sensitivity and emotional reactivity to stress, being markedly involved in the development of depressive symptoms upon exposure to stress^3^. Thus, they may also influence the degree of depression, anxiety, and other manifestations of emotional dysregulations caused by infertility-related stressors^7^. Despite this, very few studies have so far investigated the relationship between temperament and infertility or the success of infertility treatments. The sole study so far examining the direct relationship between infertility and affective temperaments found that hyperthymic temperament is protective against anxiety and depression in infertile women^66^. Likewise, no studies have examined the relationship between infertility treatment outcomes and affective temperaments so far. A few studies have already looked at the role of other personality factors in predicting IVF outcomes, some of them reporting that neuroticism may influence the success rate of in vitro fertilization (IVF)^67,68^ and exacerbate the negative emotional reactions to unsuccessful IVF outcomes^69^, and that mental disorders^70^, including anxiety, depression and distress levels, negatively impact infertility treatment success^71,72^.

In our study population of women with infertility problems, the temperament profile was already different compared to the normative population, potentially indicating a relationship between affective temperaments and infertility. There can be several reasons for this, related to relationship, couple’s attachment style, or in line with the state-of-the-art approaches to the psychological background of infertility, it might also be related to current lifestyle factors, or earlier lifestyle related factors prospectively influencing chance of getting pregnant, all of which can be affected by affective temperaments^31–34,55,73^. However, the focus of the present study is not on the psychological causes of infertility, but on how personality influences the success of treatment in cases of pre-existing infertility. In this regard, we found that among women with infertility problems, affective temperament profile was correlated with the treatment’s success, which implies that our results may have direct clinical consequences regarding fertility treatment. Although affective temperaments are relatively stable during the lifespan, they influence several modifiable risk factors of infertility, such as depression and anxiety^7^, weight, nutrition, exercise, smoking, and other health-related or self-harm behaviors^31,33^, or adherence to the treatment protocol, medication, and lifestyle changes advised by the doctor^27^, which have not been part of our present analyses. Further studies are needed to understand what mediates this affective temperament-infertility treatment outcome relationship for different temperament profiles, considering psychological pathways and processes such as emotional stability and reactivity, cognitions, and behaviors. By identifying the exact underlying mechanisms, we can later apply patient-tailored interventions, first by screening for affective temperaments in order to identify high-risk patient groups, followed by focusing on the harmful consequences of the given temperament profiles and helping them in a direct and targeted way, such as treating depression and anxiety with psychotherapeutic or pharmacological methods, strengthening adherence with closer control and education, and modifying improper lifestyle with temperament-tailored support. While we gain a deeper understanding of the complex potential mediatory relationship between affective temperaments and other targetable factors such as lifestyle or coping factors that impact infertility treatment success, our findings already give an important tool in the hands of clinical psychologists working in assisted reproduction centers, by which they can provide screening to predict who would be at a higher risk of less successful treatment, and thus will require more support in various affective temperament related behaviors, emotions, coping mechanisms and other factors to maximize their chance of benefitting from AR.

The strengths of the present study include its fair sample size and its gap-filling character through analyzing the relationship between affective temperaments and assisted reproduction treatment success, thus contributing to our knowledge about the psychological aspects of infertility. Our study also has some limitations. First, it is limited by the self-report nature of the questionnaires and the retrospective design, due to which several important factors were not assessed at the time of the treatment. Stress, depression, and anxiety levels cannot be accurately measured retrospectively, nor could certain health behaviors, like smoking, alcohol, and drug use. Therefore, these data were not included in the present analyses. Doctor’s recommendations on lifestyle changes and adherence to them were applicable only for a subgroup of the population and, therefore, are also not reported in the current paper. In the context of retrospective design, we also need to explain how we can treat it as retrospective and not cross-sectional design even though we had only one measurement point. After all, affective temperaments are relatively stable over the lifespan, so their values were approximately the same before the infertility intervention as after, so in essence we can say that we have a data point on how affective temperaments were before treatment started and then on whether the infertility treatment was successful later on. Second, the total response rate of the online questionnaires was 34,5%, which might have introduced responder bias. It is possible that those whose infertility treatment was unsuccessful responded less willingly, so they were underrepresented in the research. Also, the likeliness of responding may be associated with affective temperaments as well, which might be one possible explanation for the finding that mean affective temperament scores in our cohort were lower than the normative population average in the case of cyclothymic, anxious, and irritable temperaments. However, these normative population scores are coming from a sample smaller than our cohort (n = 438 vs 578), also their age range was completely different (between 16 and 81 years old)^36^, which makes the two samples hardly comparable. Third, clinical medical history, such as the presence of chronic or mental illnesses was self-reported by a binary yes or no answer, with no objective measures, such as HbA1c, or information about the severity of the disease. This might be the reason why, in contrast to the literature, no association was found in our cohort between metabolic disorders and infertility treatment outcomes. Fourth, while not a limitation directly, but it must be mentioned that as we considered affective temperaments as a continuous construct with a dimensional nature as is psychologically, statistically and methodologically correct, we did not used the “predominant” temperament concept, where participants are dichotomised for each temperament into nondominant temperament and dominant temperament groups, based on their score being above or below the mean + 2SD for the given temperament. While a few studies apply this approach, be believe that besides psychologically not matching the continuous distribution of affective temperaments, dichotomising a continuous variable is also a statistically faulty concept leading to loss of statistical power, inappropriate effect size, and loss of explanatory information^74^. Fifth, we aimed at using a general gynaecological sample to avoid the sample being “supernormal” in any sense, to keep our results generalizable to real life practice, which also means we did not exclude patients with psychiatric problems. While to account for this we included the presence of psychiatric disorder in the regression models as a potential confounder, we must mention that in some (especially affective disorder) patients, higher temperament scores may be related to their illness rather than to their temperaments. And lastly, the temperament of the partner was not investigated, although it may also play a role in infertility and treatment success, so future studies should consider examining the temperament of both members of the infertile couple.

## Conclusion

To the best of our knowledge, this is the first study investigating the association between affective temperaments and infertility treatment outcomes. The results suggest that affective temperaments may be related to the outcome of infertility treatments, which has clinical implications. Screening for affective temperaments may help psychologists and other health professionals working in ART centers to identify high-risk subgroups, which should, theoretically, inform the treatment plan, ideally aiding a personalized approach and enhancing the cost-effectivity of interventions, which, in turn, may help increase the chances of a successful pregnancy and live birth for women undergoing IVF treatment. Future studies should focus on how exactly affective temperaments influence infertility treatment in order to identify modifiable mediators of the effect and to increase the clinical applicability of our recent findings beyond screening potential by focusing on the harmful consequences of the given temperament profiles and helping them in a direct and targeted way.

## Data Availability

The data that support the findings of this study are available on request from the corresponding author.
